# Effects of remimazolam combined with sufentanil on hemodynamics during anesthetic induction in elderly patients with mild hypertension undergoing orthopedic surgery of the lower limbs: a randomized controlled trial

**DOI:** 10.1186/s12871-023-02249-z

**Published:** 2023-09-14

**Authors:** Qiaomin Xu, Jimin Wu, Weifeng Shan, Gongchen Duan, Haiyan Lan

**Affiliations:** Department of Anesthesia, The Sixth Affiliated Hospital of Wenzhou Medical University, No. 1188, Liyang Street, Liandu District, Lishui, 323000 Zhejiang China

**Keywords:** Remimazolam, Hypertension, Advanced age, Hemodynamics, Anesthetic induction

## Abstract

**Background:**

This randomized controlled trial was performed to observe the effect of remimazolam with sufentanil on hemodynamics during anesthetic induction in elderly patients with mild hypertension undergoing orthopedic surgery of the lower limbs.

**Methods:**

Sixty elderly patients were randomly assigned to undergo general anesthesia with intravenous injection of either remimazolam besylate (25 mg/vial, batch number 10T11011; Yichang Humanwell Pharmaceutical Co., Ltd., Yichang, China) at 0.2 mg/kg (Group R, n = 30) or propofol at 1.5 mg/kg (Group P, n = 30). Both injections were completed within 15 to 20 s. If the bispectral index value did not reach 40 to 60, then 0.05 mg/kg of remimazolam was added in Group P and 1 mg/kg of propofol was added in Group R. When the BIS value reached 40 to 60, sufentanil was administered at 0.3 to 0.5 µg/kg and cisatracurium was administered at 0.15 to 0.2 mg/kg in both groups. Three minutes later, tracheal intubation and controlled ventilation were performed to maintain the end-tidal carbon dioxide partial pressure at 4.5 to 5.0 kPa. The mean arterial pressure (MAP), heart rate (HR), cardiac output (CO), continuous cardiac index (CI), systemic vascular resistance (SVR), and pulse oxygen saturation were recorded before induction (T0), when the eyelash reflex disappeared (T1), immediately after endotracheal intubation (T2), 1 min after endotracheal intubation (T3), and 5 min after endotracheal intubation (T4). The disappearance time of the eyelash reflex, injection pain, hypotension, bradycardia, hiccupping, nausea and vomiting, and other adverse events were observed.

**Results:**

The MAP, HR, CO, and CI at T1, T2, T3, and T4 were significantly higher in Group R than P, while SVR was significantly lower in Group R than P (P < 0.05). In Group P, the MAP, HR, CO, and CI were significantly lower and the SVR was significantly higher at T1, T2, T3, and T4 than at T0 (P < 0.05). Adverse events occurred in 8 (20%) patients in Group R and 22 (73%) in Group P. The total incidence of adverse events was significantly lower in Group R than P (P < 0 0.001).

**Conclusion:**

Remimazolam combined with sufentanil for general anesthesia induction has the advantages of small hemodynamic fluctuations, stable circulation, and few adverse reactions, making it suitable for elderly patients with mild hypertension.

**Trial registration:**

Chinese Clinical Trial Registry (ChiCTR2300069224, 10/03/2023).

## Background

With the aging of the population, increasing numbers of elderly patients need surgical treatment. Hypertension is very common in elderly patients. Because elderly patients with hypertension generally have poor vascular elasticity, declining cardiovascular reserve function, and weakening of compensatory ability, hemodynamic fluctuations are likely to occur during anesthetic induction in these patients, potentially inducing serious complications. Propofol is a widely used sedative drug in the clinical setting and has the advantages of a short half-life and rapid recovery. However, the use of conventional doses of propofol in elderly patients is associated with a risk of complications such as hypotension, bradycardia, and arrhythmia [1]. Therefore, it is particularly important to reduce hemodynamic fluctuations in elderly patients and ensure their safety during anesthetic induction.

Remimazolam is a new type of anesthetic that has raised widespread concern. Remimazolam is an ultra-short-acting intravenous benzodiazepine sedative/anesthetic with the pharmacological properties of rapid onset, rapid recovery, and relatively small effects on hemodynamics [2]. Compared with propofol, remimazolam can be safely and effectively used for sedation during gastrointestinal endoscopy in elderly patients, and the incidence of sedation-related adverse reactions, especially hemodynamic events and respiratory depression, is lower [3]. Dai et al. [4] found that remimazolam was a safe and effective sedative drug during induction of general anesthesia with few adverse effects in patients with an American Society of Anesthesiologists physical status of I or II. Tang et al. [5] found that compared with propofol, remimazolam benefited cardiac surgery patients under general anesthesia by reducing hemodynamic fluctuations. They reported that remimazolam influenced the surgical stress response and respiratory function, thereby reducing anesthesia-related adverse reactions. Liu et al. [6] found that remimazolam may have non-inferior efficacy and a higher safety profile than etomidate plus propofol in elderly outpatients undergoing colonoscopy, which suggests that remimazolam may be more suitable for elderly outpatients undergoing colonoscopy. Therefore, we speculate that remimazolam may be a better choice for sedation in elderly patients and is expected to become the preferred sedative in clinical practice. In this study, the effect of remimazolam combined with sufentanil on hemodynamics during anesthetic induction was evaluated in elderly patients with mild hypertension undergoing orthopedic surgery of the lower limbs.

## Methods

### Clinical data

This study was approved by the Medical Ethics Committee of The Sixth Affiliated Hospital of Wenzhou Medical University in Lishui, China (LLW-FO-403) and was registered at the Chinese Clinical Trial Registry (ChiCTR2300069224, 10 March 2023). Written informed consent was obtained from all patients. All methods were carried out in accordance with relevant guidelines and regulations.

**Fig. 1:**
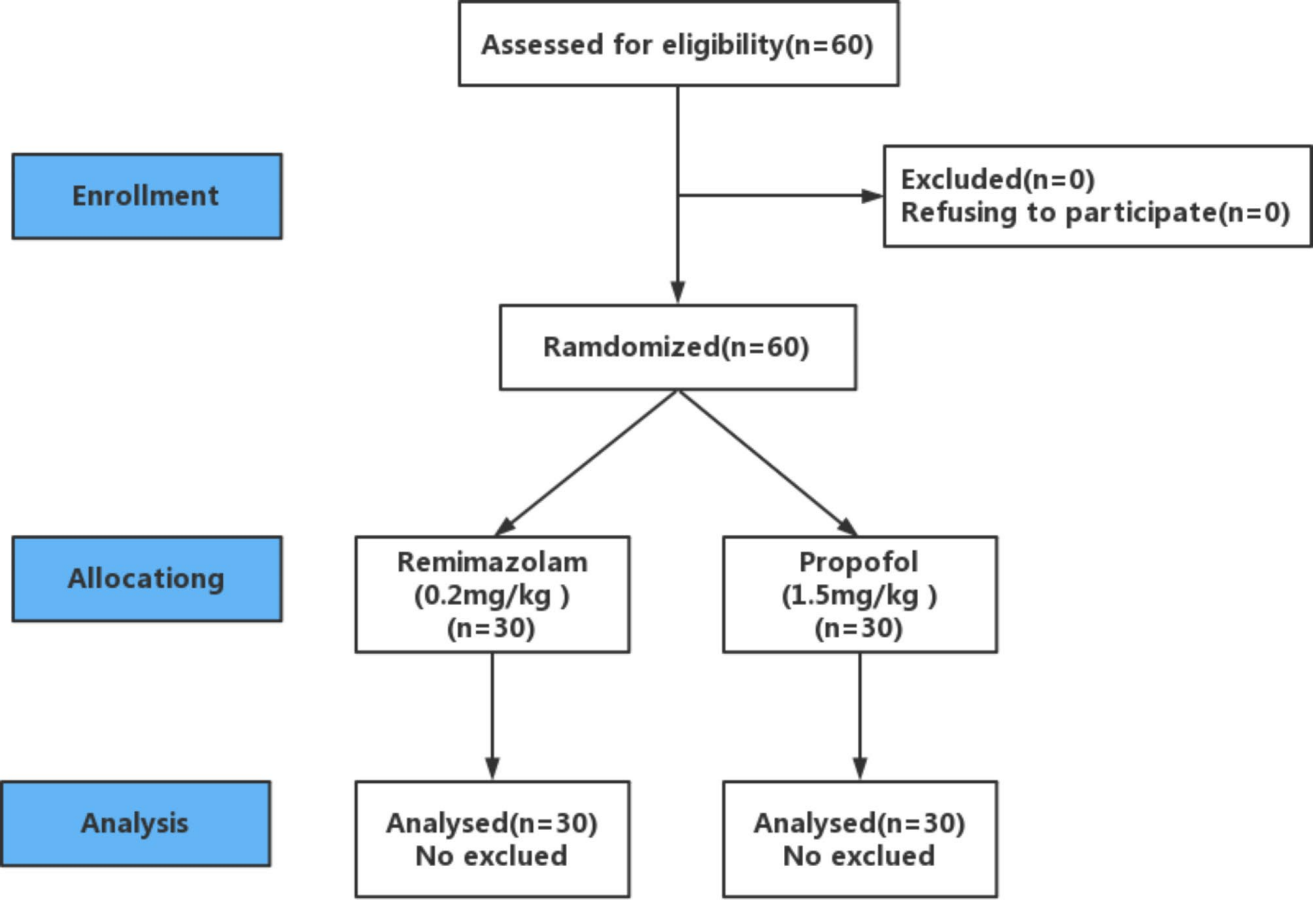
Flow of participants through the study

After obtaining approval from the hospital’s medical ethics committee and written consent from the patients, we enrolled 60 elderly patients with mild hypertension who underwent elective orthopedic surgery of the lower limbs from August 2021 to March 2022. The selected patients met the following criteria: 65 to 75 years of age, American Society of Anesthesiologists physical status of II or III, cardiac function class I or II, and blood pressure controlled within 160/90 mmHg after standardized treatment for those with a history of essential hypertension. None of the patients had serious arrhythmia or serious liver and kidney dysfunction, and none had a history of mental illness. The exclusion criteria were hypertension without regular medical therapy, bradycardia, and second-degree or worse atrioventricular block. The patients enrolled in the study comprised 27 male and 33 female patients with an age of 65 to 80 years and body weight of 50 to 78 kg. According to the order of treatment, the patients were randomly divided into two groups of 30 patients each: remimazolam 0.2 mg/kg (Group R) and propofol 1.5 mg/kg (Group P) (Fig. 1).

### Methods

The patients in both groups were routinely administered 10 mL/kg/h lactated Ringer’s solution in a readily accessible hand vein upon admission, and the mean arterial pressure (MAP), electrocardiogram, heart rate (HR), pulse oxygen saturation (SpO_2_), and bispectral index (BIS) value were continuously monitored with a multifunctional monitoring device (ConView YY-106; PearlCare, Hangzhou, China). Upon completion of monitoring, a noninvasive continuous arterial blood pressure and hemodynamic monitoring system (TL-400; Zhejiang Shanshi Biological Pharmaceutical Equipment Co., Ltd., Hangzhou, China) was used to monitor the patients’ hemodynamic indexes, and efforts were made to relieve any nervousness. The baseline HR and MAP were defined as the average of three HR and MAP measurements before the procedures. All patients were given oxygen via a face mask (6 L/min) before the induction of anesthesia. Remimazolam besylate (25 mg/vial, batch number 10T11011; Yichang Humanwell Pharmaceutical Co., Ltd., Yichang, China) was intravenously administered at 0.2 mg/kg in Group R, and propofol (20 mL, 0.2 g, batch number HJ20150654; Fresenius Kabi Austria GmbH, Graz, Austria) was intravenously administered at 1.5 mg/kg in Group P. Both injections were completed within 15 to 20 s. If the BIS value did not reach the range of 40 to 60, then 0.05 mg/kg of remimazolam was added in Group P and 1 mg/kg of propofol was added in Group R. In both groups, controlled ventilation was performed after the eyelash reflex disappeared. When the BIS value reached 40 to 60, sufentanil was administered at 0.3 to 0.5 µg/kg (50 µg/vial, batch number 10A11141; Yichang Humanwell Pharmaceutical Co., Ltd.) and cisatracurium was administered at 0.15 to 0.2 mg/kg in both groups. Three minutes later, tracheal intubation and controlled ventilation were performed to maintain the end-tidal carbon dioxide partial pressure at 4.5 to 5.0 kPa. Anesthesia was maintained with propofol at 3 to 5 mg/kg/h, remifentanil at 10 to 15 µg/kg/h, and 1–2% sevoflurane; cisatracurium was intermittently injected, and the BIS value remained at 40 to 60. Sevoflurane, propofol, and remifentanil were discontinued during skin suturing. The disappearance time of the eyelash reflex and hemodynamic indices including MAP, HR, cardiac output (CO), continuous cardiac index (CI), and systemic vascular resistance (SVR) were measured at five different time points: before induction (T0), when the eyelash reflex disappeared (T1), immediately after endotracheal intubation (T2), 1 min after endotracheal intubation (T3), and 5 min after endotracheal intubation (T4).

Adverse events were defined as follows [5]. Hypotension was defined as a > 30% decrease in systolic blood pressure from baseline or MAP of < 65 mmHg during induction. In such cases, 5 µg norepinephrine was administered until the systolic blood pressure was > 70% of baseline or the MAP was ≥ 65 mmHg. Atropine 0.5 mg was intravenously injected if the HR decreased to < 50 beats/minute. Injection site pain was detected by a withdrawal response or a numeric rating scale score of ≥ 3 (where 0 indicated “no pain” and 10 indicated “unbearable pain”).

### Outcomes

#### Primary outcomes

The primary outcomes of this study were the disappearance time of the eyelash reflex and the following hemodynamic indices: MAP, HR, CO, CI, and SVR.

#### Secondary outcome

The secondary outcome of this study was the incidence of adverse events, including hypotension, bradycardia, hiccupping, nausea, injection pain, and vomiting. The patients were monitored for these events from the beginning of anesthetic induction to the completion of endotracheal intubation.

### Statistical analysis

The sample size was estimated using PASS 11 software (NCSS, LLC, Kaysville, UT, USA). In our pilot study, the incidence of hypotension elicited by 1.5 mg/kg of propofol was 40% (8/20), which was reduced to 10% (2/20) after pretreatment with 0.2 mg/kg of remimazolam. To achieve 80% (β = 0.20) statistical power with α = 0.05, each group would require no less than 23 patients. To account for drop-outs, we recruited 30 patients per group.

Data processing was performed using SPSS 15.0 (SPSS Inc., Chicago, IL, USA). The normality test in SPSS was used to determine whether the data conformed to a normal distribution. Measurement data are expressed as mean ± standard deviation. Repeated-measures analysis of variance was performed for intragroup comparisons, and one-way analysis of variance was performed for intergroup comparisons. A P value of < 0.05 was considered statistically significant.

## Results

### Demographic characteristics

This trial was conducted from August 2021 to March 2022. Sixty eligible patients were screened and included in the final analyses. The patients’ demographic characteristics, including sex, age, and body mass index, were similar between the two groups (P > 0.05) (Table 1).

**Table 1:** Demographic data

Group	Number of cases (n)	Sex ratio (male/female)	Age (years)	Body mass index
Group R	30	14/16	69.9 ± 4.3	21.5 ± 2.3
Group P	30	13/17	68.6 ± 3.3	22.2 ± 2.4
*P value*		0.795	0.194	0.254

Values are expressed as mean ± standard deviation or number of cases. No statistically significant differences in sex, age, or body mass index were observed between the groups.

### Comparison of hemodynamics between the two groups before and after induction

There were no significant differences in the MAP, HR, SpO_2_, CO, CI, or SVR between the two groups at T0 (P > 0.05). The MAP, HR, CO, and CI at T1, T2, T3, and T4 were significantly higher in Group R than P, while SVR was significantly lower in Group R than P. In Group P, the MAP, HR, CO, and CI at T1, T2, T3, and T4 were significantly lower than those at T0, while the SVR at T1, T2, T3, and T4 was significantly higher than that at T0 (P < 0.05) (Table 2).

**Table 2:** Hemodynamic data

Item	Group	T0	T1	T2	T3	T4
MAP(mmHg)						
	Group R	102.7 ± 8.6	98.3 ± 6.5	100.7 ± 8.5	99.3 ± 7.4	98.5 ± 7.9
	Group P	103.8 ± 7.7	71.3 ± 6.2^a*^	86.7 ± 7.7^a*^	82.3 ± 7.3^a^	81.7 ± 7.6^a^
*P value*		0.604	**< 0.001**	**< 0.001**	**< 0.001**	**< 0.001**
HR(bpm)						
	Group R	78.9 ± 9.5	75.7 ± 8.5	76.9 ± 9.3	76.5 ± 8.9	75.9 ± 8.6
	Group P	77.5 ± 8.9	59.8 ± 9.4^a*^	63.7 ± 8.9^a*^	65.8 ± 8.4^a*^	64.9 ± 9.1^a*^
*P value*		0.558	**< 0.001**	**< 0.001**	**< 0.001**	**< 0.001**
SP0_2_(%)						
	Group R	95.7 ± 1.5	98.2 ± 0.9	99.2 ± 0.4	99.7 ± 0.6	99.8 ± 0.4
	Group P	96.3 ± 1.2	98.6 ± 0.7	99.4 ± 0.6	99.5 ± 0.7	99.6 ± 0.6
*P value*		0.093	0.060	0.134	0.240	0.134
CO(L/min)						
	Group R	4.5 ± 0.7	4.2 ± 0.6	4.4 ± 0.6	4.3 ± 0.5	4.4 ± 0.6
	Group P	4.6 ± 0.6	3.2 ± 0.6^a*^	3.4 ± 0.7^a*^	3.6 ± 0.6^a*^	3.6 ± 0.5^a*^
*P value*		0.555	**< 0.001**	**< 0.001**	**< 0.001**	**< 0.001**
CI(L/min/m^2^)						
	Group R	3.5 ± 0.9	3.3 ± 0.6	3.4 ± 0.5	3.4 ± 0.4	3.4 ± 0.6
	Group P	3.6 ± 0.8	2.6 ± 0.7^a*^	2.7 ± 0.6^a*^	2.9 ± 0.3^a*^	2.9 ± 0.4^a*^
*P value*		0.651	**< 0.001**	**< 0.001**	**< 0.001**	**< 0.001**
SVR(dyn·s/cm^5^)						
	Group R	990 ± 110	1006 ± 129	1115 ± 124	1166 ± 127	1105 ± 124
	Group P	985 ± 116	2556 ± 136^a*^	2010 ± 128^a*^	1955 ± 131^a*^	1943 ± 121^a*^
*P value*		0.865	**< 0.001**	**< 0.001**	**< 0.001**	**< 0.001**

Data are presented as mean ± standard deviation

^a^P<0.05 compared with T0

*P < 0.05 compared with Group P

MAP: mean arterial pressure; HR: heart rate; SpO_2_, pulse oxygen saturation; CO: cardiac output; CI: cardiac index; SVR: systemic vascular resistance

### Comparison of eyelash reflex disappearance time and adverse events between the two groups

The disappearance time of the eyelash reflex was significantly longer in Group R than P. Adverse events occurred in 8 (20%) patients in Group R and 22 (73%) in Group P. The total incidence of adverse events was significantly lower in Group R than P (P < 0.001). There was no significant difference in hiccupping or nausea and vomiting between the two groups (P > 0.05) (Table 3).

**Table 3:** Disappearance time of eyelash reflex and incidence of adverse events

Group	Disappearance time of eyelash reflex (seconds)	Injection pain	Hypotension	Bradycardia	Hiccup	Nausea and vomiting	Total incidence of adverse events
Group R	63.4 ± 6.5	1(3)	1(3)	4(13)	2(7)	0(0)	8(20)
Group P	29.4 ± 7.2^*^	22(73)^*^	19(63)^*^	16(53)^*^	0(0)	0(0)	22(73)
*P value*	**< 0.001**	**< 0.01**	**< 0.01**	**< 0.01**	0.472		**< 0.01**

Data are presented as mean ± standard deviation or number (percentage) of patients

^*^P < 0.05 compared with Group R

## Discussion

In this study, we demonstrated that the MAP, HR, CO, and CI at T1, T2, T3, and T4 were significantly higher in Group R than P, while SVR was significantly lower in Group R than P. The incidence of total adverse events was 73% in patients in Group P but only 20% in patients in Group R.

Elderly patients’ health is often complicated by a variety of diseases, of which hypertension is the most common. Their tolerance to anesthesia and surgery is lower than that of younger patients. Severe hemodynamic fluctuations can easily induce cerebrovascular accidents. Tracheal intubation during anesthetic induction is a severely noxious stimulus and can have a severe impact on patients’ hemodynamics. Maintaining stable hemodynamics of elderly patients during induction and improving the blood perfusion of the heart, brain, kidney, and other important organs can improve the disease outcome and prognosis of elderly patients [7, 8]. How to safely navigate the induction period for elderly patients with complications has been a topic of debate among scholars worldwide.

As a commonly used anesthetic in the clinical setting, propofol has the advantages of rapid onset, short duration of action, no accumulation in the body, and minimal postoperative nausea and vomiting. Propofol is widely used in the induction and maintenance of anesthesia. It exerts its sedative and hypnotic effects through interaction with gamma-aminobutyric acid receptors and has certain cardiovascular inhibitory effects that can lead to a decrease in blood pressure, CO, and HR [9–11]; these effects were also confirmed in the present study. Severe circulatory depression during induction can lead to insufficient blood perfusion of the heart, brain, kidneys, and other important organs, leading to serious complications such as myocardial infarction and cerebral infarction, which seriously affect the prognosis of elderly patients. The BIS primarily relies on electroencephalography to indicate the depth of consciousness during anesthesia. It is the first detection index approved and certified by the U.S. Food and Drug Administration and has a good correlation with the depth of sedation. The BIS is used to monitor the degree of brain inhibition and judge the depth of sedation, and it has a good correlation with the depth of sedation with propofol [12, 13]. The BIS is thus widely used in the clinical setting. Flumazenil is an imidazobenzodiazepine that promptly reverses the hypnotic and sedative effects of benzodiazepines via competitive inhibition of gamma-aminobutyric acid receptors. One study demonstrated that flumazenil given to healthy, unpremedicated patients during propofol/remifentanil anesthesia significantly increased the BIS value [14]. None of the patients in the present study received flumazenil, and the BIS value was accurate and reliable.

Remimazolam is a new benzodiazepine drug that has the advantages of rapid recovery and metabolism, no drug accumulation, independence from liver and kidney metabolism, and few side effects [15–19]. However, few studies have used remimazolam for anesthetic induction in elderly patients with mild hypertension. The present study showed that the effect of propofol on hemodynamics in elderly patients during induction was greater than that of remimazolam; specifically, blood pressure decreased, HR decreased, continuous CO and CI decreased, and SVR increased (P < 0.05). These findings indicate that propofol induces significantly greater cardiovascular inhibition than remimazolam. This study also showed that these two drugs combined with sufentanil can effectively inhibit intubation reactions and can be used in anesthetic induction of elderly patients with mild hypertension, but remimazolam has a slightly weaker effect on the circulation. However, the sample size of this study was small, and a single drug dose and induction method was used in both groups of patients. Thus, anesthetic induction for elderly patients, including selection of appropriate drugs and doses and formulation of an appropriate anesthetic induction regimen, should be performed on an individual basis according to each patient’s specific situation.

## Conclusions

Like propofol, remimazolam can also be used for anesthetic induction in elderly patients. The injection pain was significantly less severe with remimazolam than with propofol, thus improving patients’ comfort. Remimazolam combined with sufentanil for general anesthesia induction produces minimal hemodynamic fluctuation, stable circulation, and few adverse reactions, making it suitable for elderly patients with mild hypertension.

## Data Availability

The original contributions presented in this study are included in the article. Further inquiries can be directed to the corresponding authors.
